# Supplementary material for: Annealing Effects on SnO2 Thin Film for H2 Gas Sensing
Source: Nanomaterials (Basel). 2022 Sep 16;12(18):3227. doi: 10.3390/nano12183227 (PMC9501209; doi:10.3390/nano12183227)
Supplement: Supplementary file 1 [file nanomaterials-12-03227-s001.zip › nanomaterials-1914334-supplementary.pdf]

nanomaterials

Supplementary material for

Annealing Effects on SnO_2 Thin Film for H_2 Gas Sensing

Yijun Yang [1,†], Bohee Maeng [1,†], Dong Geon Jung [1], Junyeop Lee [1,2], Yeongsam Kim [1], JinBeom Kwon [1], Hee Kyung An [1] and Daewoong Jung [1,*]

[1] Advanced mechatronics R&D Group, Korea Institute of Industrial Technology (KITECH), Daegu 42994, Korea
[2] School of Electronic and Electrical Engineering, College of IT Engineering, Kyungpook National University, 80 Daehakro, Daegu 41566, Korea
* Correspondence: dwjung@kitech.re.kr; Fax: +82-53-580-0160
† These authors contributed equally to this work.

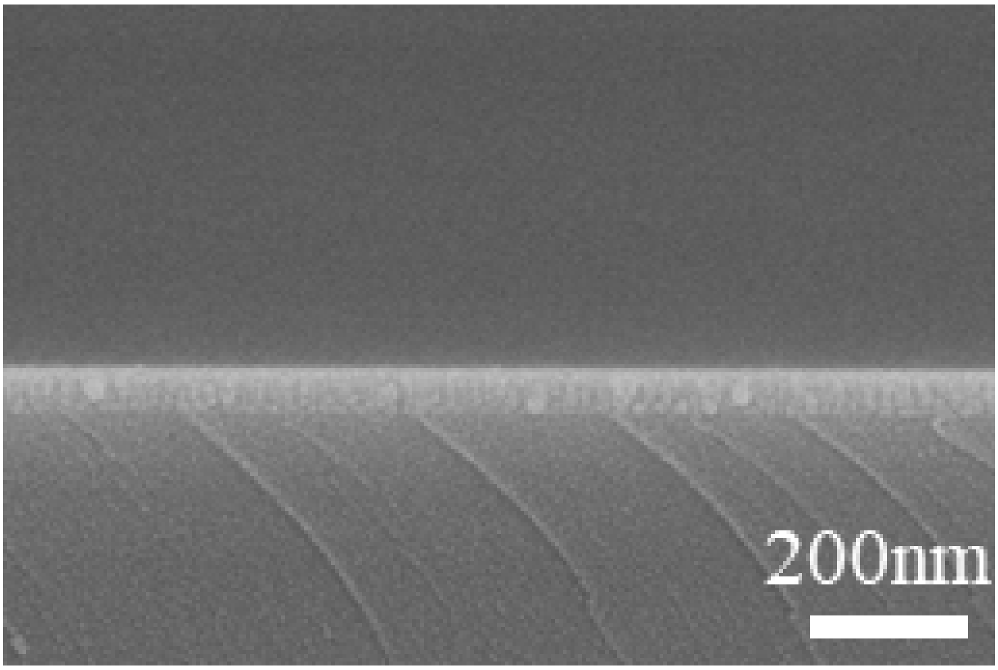

Figure S1. Cross-section SEM image of unannealed SnO_2.

The thickness of film which is not annealed SnO2 is about 55.6 nm by SEM cross-section image.

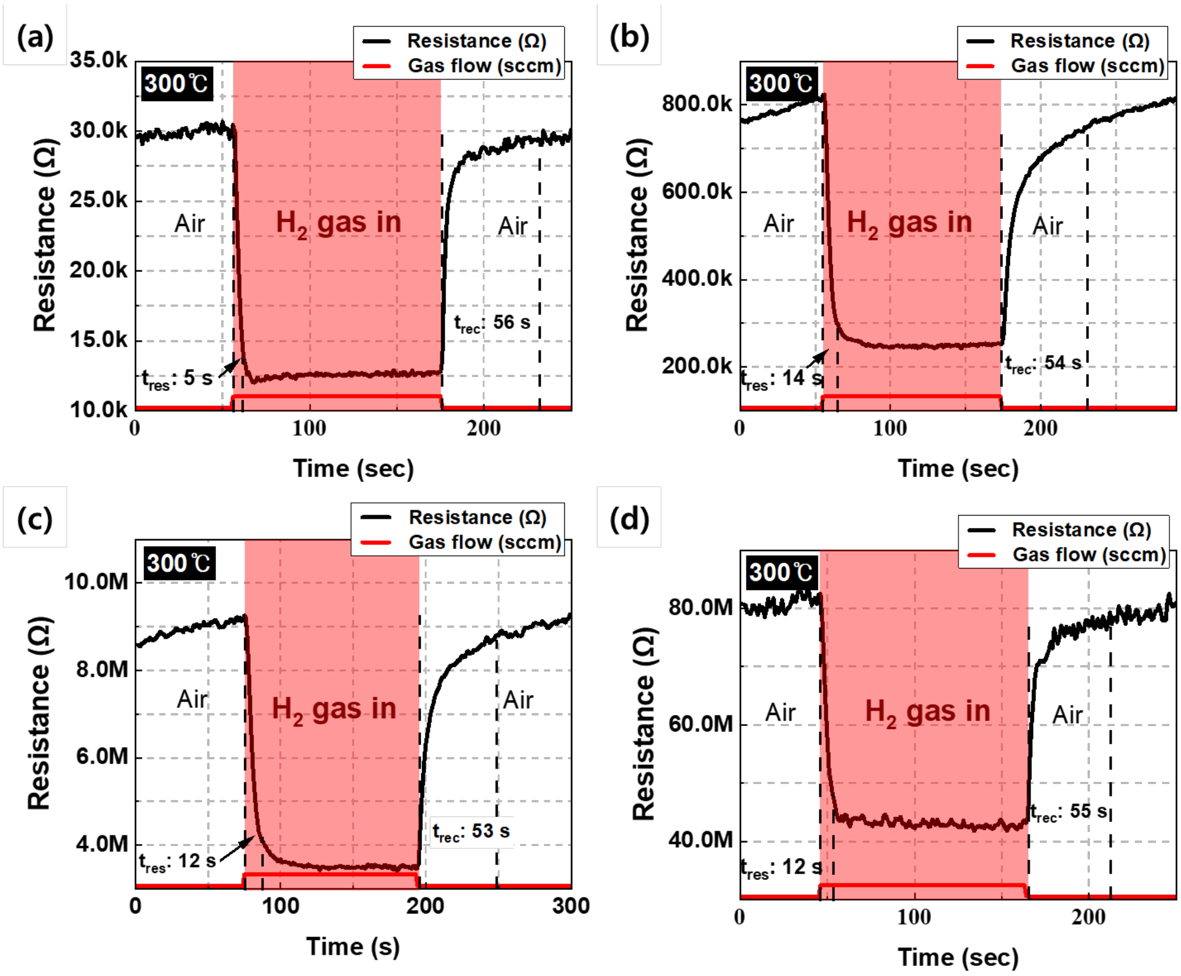

Figure S2. Hydrogen response and recovery time of (a) unannealed SnO_2 (b) 1-hour annealed SnO_2 (c) 3-hour annealed SnO_2 (d) 5-hour annealed SnO_2.

The resistance of the film is increased for annealing time at 300°C indicating about 35k, 800k, 9M, and 80M for not-annealed, 1-h, 3-h, and 5-h each. As annealing temperature increases, the initial resistance of SnO_2 increases for some point, but after the inflection point it decreases [1].

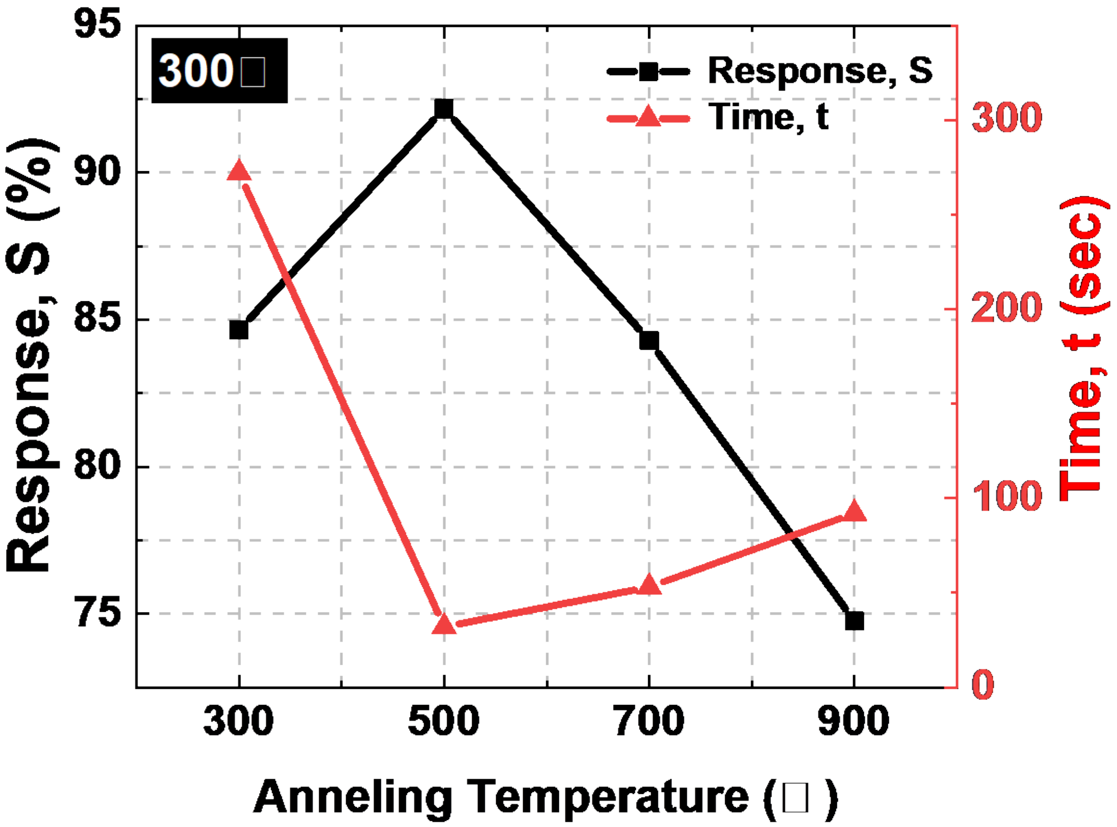

Figure S3. Hydrogen response and time for annealing temperature.

References

[1] Kang, K.M.; Choi, J.U. Growth characteristic of SnO2 thin film for gas sensor with annealing treatment, J. Kor. Inst. Surf. Eng. 2007, 6, 258-261.
